# The Structural Proteins of Membrane Rafts, Caveolins and Flotillins, in Lung Cancer: More Than Just Scaffold Elements

**DOI:** 10.7150/ijms.87836

**Published:** 2023-10-02

**Authors:** Ana Karina Saldaña-Villa, Roberto Lara-Lemus

**Affiliations:** Department of Molecular Biomedicine and Translational Research, Instituto Nacional de Enfermedades Respiratorias “Ismael Cosío Villegas”. Mexico City, Mexico.

**Keywords:** Lung cancer, membrane rafts, caveolins, flotillins, carcinogenesis.

## Abstract

Lung cancer is one of the most frequently diagnosed cancers worldwide. Due to its late diagnosis, it remains the leading cause of cancer-related deaths. Despite it is mostly associated to tobacco smoking, recent data suggested that genetic factors are of the highest importance. In this context, different processes meaningful for the development and progression of lung cancer such endocytosis, protein secretion and signal transduction, are controlled by membrane rafts. These highly ordered membrane domains contain proteins such as caveolins and flotillins, which were traditionally considered scaffold proteins but have currently been given a preponderant role in lung cancer. Here, we summarize current knowledge regarding the involvement of caveolins and flotillins in lung cancer from a molecular point of view.

## Introduction

Primary lung cancer (LC) is the leading cause of cancer death worldwide. In 2020, it was the most diagnosed cancer after breast cancer 1,2. Non-small cell lung cancer (NSCLC) is the most frequent type of LC, accounting for approximately 80% to 85% of the cases, while small cell lung cancer (SCLC) accounts for approximately 15% of all the patients 3. LC is associated with tobacco smoking, but recent data indicate that 14% of LC deaths can be attributed to air pollution exposure; these values vary between 5% to more than 20% in different countries 4. These data imply that LC currently affects populations with low smoking prevalence.

LC is influenced by endogenous and external factors that impact different molecular mechanisms involved in cell division, differentiation, and death, eventually leading to cellular transformation, cancer, and metastasis. LC have significant differences in cellular morphology, and despite progress in treatment approaches, the overall survival rate within five years is still less than 20% 5,6. Therefore, it is crucial to understand better the molecular mechanisms underlying LC development, progression, and metastasis in order to improve prevention, treatment efficacy, and survival.

Recent advances in cancer research have revealed the complex scenario, and dynamic nature of cancer pathogenesis. In particular, research in epigenetics has evidenced that cancer involves profound changes in cellular functions due to alterations in genome structure, stability, and gene expression 7-9. Growth receptors, transducing mediators, and effectors play a crucial role in regulating cell proliferation, and significant advances have been reported regarding the contribution of these molecules in carcinogenesis, tumor progression, and drug targeting 10-12.

Lipid rafts (LR) are membrane subdomains involved in the transport and localization of different signaling transduction elements regulating cell proliferation. For example, it is a well-documented fact, that LR participates in the translocation of the tumor necrosis factor receptor, which is essential for TNF-α-mediated NF-κB activation. Disturbing LR organization switches the outcome of TNF-α signaling from NF-κB activation to apoptosis 13. However, the spatiotemporal organization of this signaling network is incompletely understood. Current knowledge states that the endoplasmic reticulum delivers secreted and membrane proteins to the Golgi apparatus through transport vesicles. After they are properly folded and modified, mature proteins are transported into the plasma membrane (PM) or different organelles through endosomal, regulated, or constitutive secretory pathways. Distinct sorting proteins regulate cellular trafficking, and malfunctioning of this process causes diseases, including cancer 14-16. In this review, we will focus on recent data about the role of LR-structural proteins, caveolins, and flotillins in LC. These proteins are important because the appropriate assembly of membrane subdomains partly determines the functionality of several proteins implied in cancer. Based on the experimental work performed in LC and other types of cancer, we propose a rationale to explore the role of LR-associated proteins in cancer development.

## The role of MR proteins in LC

LR are small (ranging from 10 to 200 μm), specialized dynamic cellular membrane subdomains. Currently, the term membrane rafts (MR) is preferred 17, and two distinct types are recognized based on the density of their packing and chemical nature: planar and flask-shaped or caveolae. MR are composed of sphingolipids, mainly sphingomyelin and gangliosides 18, cholesterol 19, and particular proteins (proteolipids). MR are resistant to solubilization by non-ionic detergents (DRMs) 20; this allows its isolation from cellular membranes at low temperature 21-23. However, DRM are MR aggregates and do not actually represent the native state of MR in living cells 24. The harmonious interactions between lipids and lipid-associated proteins determine MR structure and function. Therefore, the traditional view of MR proteins as “scaffold supports” has been replaced by the recognition that these proteins organize and control several cell membrane attributes 25,26. Ordered aggregation of homologous MR through protein-protein or lipid-protein interactions can stabilize larger structures functioning as platforms, which could include or exclude particular proteins for specific biochemical processes. MR have diverse cellular functions and are associated with a growing number of specialized functions at the PM level. However, the functions of these structures in subcellular membrane compartments, such as the Golgi, endocytic endosomes or exosomes, are not fully understood. Moreover, since cholesterol is synthesized in the endoplasmic reticulum (ER) but sphingolipids are synthesized in the Golgi, the first MR assemblage largely occurs in Golgi membranes 27,28, and MR participates in the subsequent delivery of transport vesicles and recycling pathways 29.

The PM plays a key role in signal transduction, and a close association between cell signaling processes and MR has been well documented. Many proteins involved in cell signaling, such as receptors, GTP-binding proteins, kinases, and phosphatases, can be selectively incorporated into the membrane in response to endogenous or exogenous stimuli. This is possible because of the asymmetric structure of both PM leaflets and the physicochemical properties conferred by the liquid-ordered phases of the MR. Currently, there is a general agreement that individual MR domains can harbor only a few proteins; thus, MRs must cluster together 29. Raft clustering on the extracellular face of PM can be triggered by different molecules like antigens, antibodies, or other MR-binding proteins, including cholera toxin B. 30. Cytoplasmic leaflet proteins such as flotillins and annexins could serve as clustering agents 31,32. In addition, the recruitment and concentration of death receptors could also aggregate MR, forming clusters of apoptotic signaling molecule-enriched rafts (CASMERs). CASMERs favor the activation of procaspase 8 and apoptosis through downstream signaling molecules 33. Finally, it is important to emphasize that the dynamic changes in MR composition could guarantee the proper chemical modification of signal transducers and protect MR resident proteins from degradation. Therefore, the dynamic MR composition would ensure the functionality of PM transducers in signaling pathways 34,35.

Several proteins are structurally and functionally related to MR in both physiological and pathological states. In fact, a comprehensive MR-proteomic database providing information about this topic is available (Mohamed A. et al. 36). MR-associated proteins include caveolins, which are found in caveolae, and flotillins and raft-linking proteins (raftlins), which are found in flat rafts 37-39. These proteins have important roles in cancer development and the regulation of tumor microenvironment and metastasis.

### Caveolins

Caveolae are 50-100 nm flask-shaped plasma membrane domains enriched in lipids such as ceramide, phosphatidic acid, diacylglycerides, and glycosphingolipids 40. Caveolae are involved in different physiological processes, including transport, cell survival, proliferation, migration, and apoptosis. Since caveolae were first described 41, several reports have depicted roles for caveolae in human pathologies 42,43. The main structural proteins of caveolae are a family of proteins known as caveolins. These proteins play an important role in the pathogenesis of several diseases, including different types of carcinomas.

So far, the role of caveolin 1, (cav 1) is the best characterized in LC. Cav 1 is a 22 kDa MR-associated protein involved in different normal cell functions, including lipid transportation, cell growth, and death regulation. Different studies support that cav 1 may have opposite roles: it may act as a tumor suppressor and a cancer promoter. The role of cav1 in cancer depends on the cell type and cancer stage 42-44. In spite of *CAV1* expression is mostly reduced in LC cell lines 45,46, it was expressed in 76% of NSCLC cell lines compared with normal human lung epithelial cells. Moreover, silencing *CAV1* in NSCLC cells inhibited cell proliferation and colony formation. However, *CAV1* was downregulated in 95% of SCLC cell lines, and re-expression of cav1 in SCLC resulted in a decreased capacity to form cell colonies 47. These findings account for a differentiated role of this protein: as an oncoprotein in NSCLC and as a tumor suppressor in SCLC. Other lines of evidence from *in vivo*, *in vitro*, and clinical studies have shown that *CAV1* expression may account for anti-tumor properties or the aggressiveness of carcinomas 48-54. Caveolae are important for regulating cancer signaling because they hold different signaling molecules 55. Cav 1 and other MR-associated proteins act as oncoproteins or tumor suppressors by activating cell proliferation and survival pathways or by blocking specific components of transduction cascades. For example, residues 82-101 in cav 1-N-terminal domain (caveolin scaffolding domain, CSD) interact with many signaling proteins, such as the nonfunctional forms of endothelial nitric oxide synthase (soluble form of cav 1), heterotrimeric G proteins, and MAP kinase, to negatively regulate their activation 36,56-58.

#### The role of cav 1-tyrosine 14

**Figure 1:**
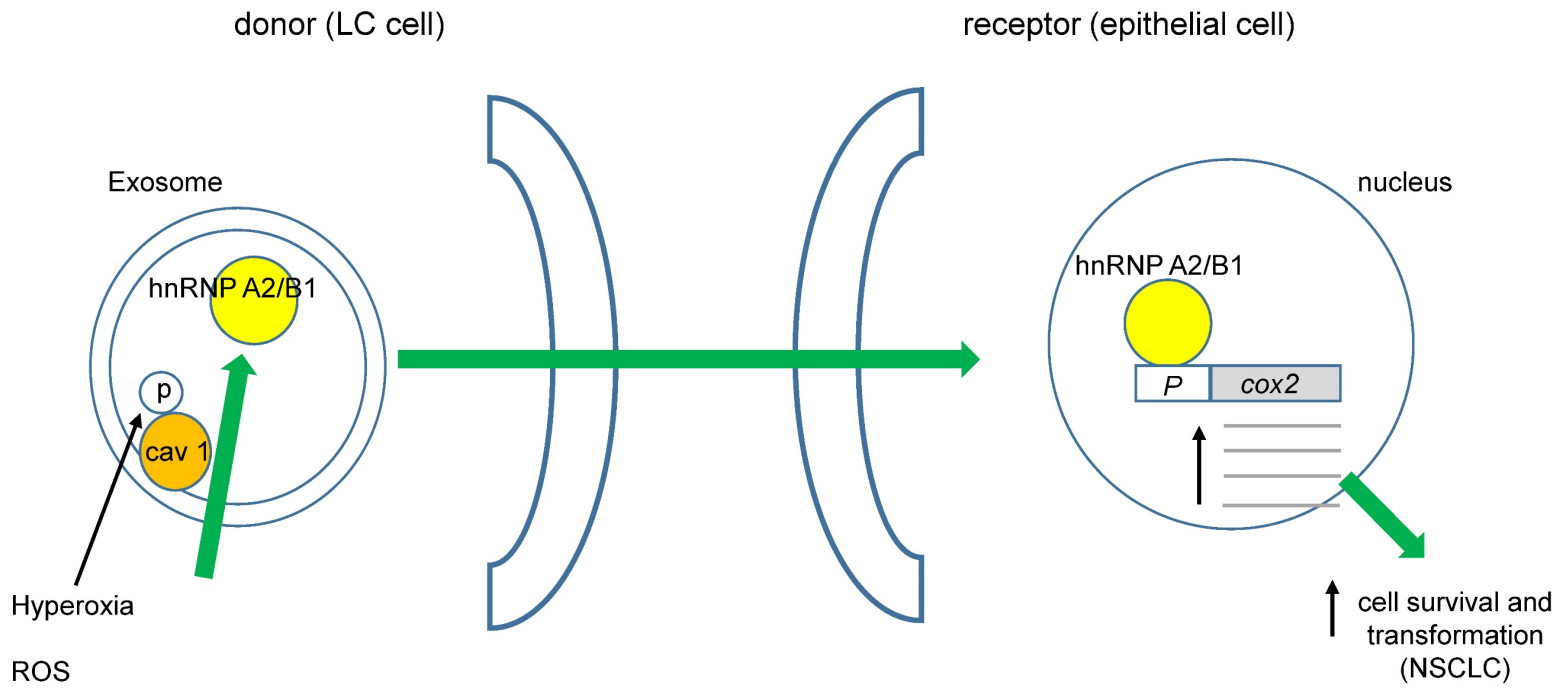
** ROS could induce cell transformation in NSCLC.** Phosphorylation of cav 1-Y14 increases load of hnRNAPA2/B1 into exosomes, they move towards non-transformed epithelial cells favoring its transformation through enhanced *cox 2* transcription.

**Figure 2:**
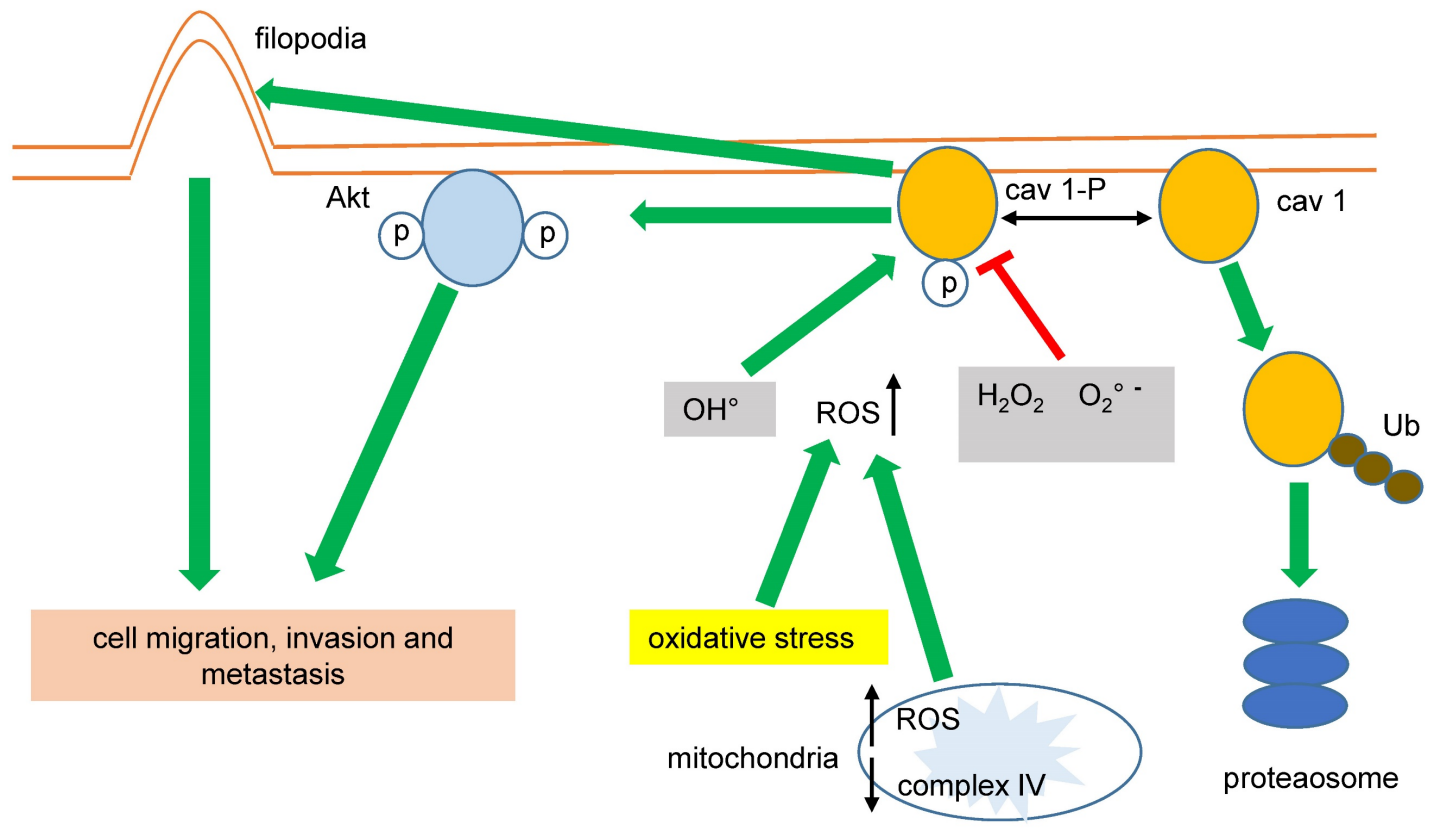
** Phosphorylation of cav 1-Y14 and its oncogenic activities in LC is differentially regulated by ROS.** Superoxide anion and hydrogen peroxide promote Y14 dephosphorylation and cav 1 proteasome degradation, whereas hydroxyl radical provoke enhanced cell migration, invasion and metastasis.

It was recently demonstrated that cav 1 phosphorylated at tyrosine 14 (pY14) becomes exacerbated in exosome membranes (microvesicles, MVs) during cell-hyperoxia and the production of reactive oxygen species (ROS). This increase in pY14 cav1 resulted in a higher release of the heterogeneous nuclear ribonucleoproteins A2/B1 (hnRNPA2/B1) into MVs 59. Furthermore, hnRNPA2/B1 specifically binds to the COX-2 core promoter, favoring NSCLC 60, (figure 1). Interestingly, ROS also could directly affect cav 1's role in cell migration of human LC cells by activating Akt (protein kinase B) through phosphorylation of Akt-T308 or S473. However, opposite effects of superoxide anion and hydrogen peroxide with respect to the hydroxyl radical were observed 61. Several studies have demonstrated that oxidative stress induces the phosphorylation of cav 1-Y14, increasing cell migration. Recently, Jiang and coworkers found that cav 1- pY14 restricts the mitochondrial recruitment of mitofusin 2 in breast cancer cells. Since mitofusin 2 is critical for mitophagy, this process results in ROS accumulation and damaged mitochondria 62. Moreover, hydrogen peroxide favors the phosphorylation of cav 1-Y14. These data point to a reciprocal interaction between oxidative stress and cav 1 function (figure 2).

Impaired mitochondrial function and increased glycolysis (Warburg effect) are hallmarks of cancer cells. In this sense, the expression of *CAV1* was greatly correlated with higher glycolysis rates and the inhibition of the mitochondrial complex IV in MDA-MB-231 breast cancer cells. Conversely, the inhibition of the mitochondrial complex IV increased the mitochondrial levels of ROS associated with cav 1-pY14, favoring cell migration and invasion 61,63, (figure 2).

Cav 1 improves the migration of cancer cells by other mechanisms; in lung adenocarcinoma (LUAD) cells, cav 1 increases invasion favoring both filopodia and lamellipodia formation 64,65. Downregulating *CAV1* gene expression by shRNA-cav 1-transduction into NSCLC H460 and H292 significantly decreased cell migration and the expression of integrins β1 and β3 66. Again, these data suggest that cav1-pY14 promotes cancer cell migration, invasion, and metastasis, (figure 2). Interestingly, cav 1-Y14 could also link cell mobility, migration, invasion, and oxidative stress in LC because overexpression of cav 1-Y14F displayed a dominant negative phenotype, similar to the cav 1 RNAi-mediated knockdown 67.

Another recent report based on bioinformatics identified a novel connection between cav 1 and cell motility. Cav 1 modulated the activity of cellular calcium fluxes, and both cav 1 and c-Myc displayed a favorable effect on the O-GlcNAcylation. Since O-GlcNAcylation coordinates calcium signaling via the TRPM7 channel, this process increased cell migration and invasion in LC cell lines 68. Finally, as mentioned previously, cav 1 regulates several signal transduction pathways. Activating MR-resident proteins such as epidermal growth factor receptor (EGFR) increased the proliferation, migration, invasion, and volume of xenograft tumors, both in human lung tumor tissues and the human LUAD cell line GLC-82 69. siRNA-mediated downregulation of cav 1 caused a stable proliferation arrest in human LUAD and SCLC cell lines, showing a substantial decrease of pAkt and its downstream effectors, phosphorylated ERK (pERK) and STAT3 (pSTAT3). These findings highlight that the tumorigenic effects displayed by the overexpression of cav 1 are partly mediated by the EGFR/ERK signaling pathway 70.

#### Genetic and epigenetic regulation of cav 1 and LC

##### Promoter hypermethylation

Cancer development is associated with hypermethylation of specific cytosine residues of the CpG dinucleotide located in or near gene promoters (CpG-islands), global hypomethylation, and selective demethylation of the regulatory regions of some genes 71,72. Promoter methylation and acetylation/deacetylation of histones are common epigenetic mechanisms that lead to activation/repression of transcription of several genes, mainly tumor suppressor genes. In some types of cancer, including LC, DNA-methylation represses the *CAV1* gene at cancer onset, but demethylation and the concomitant re-expression of this gene occur before metastasis 73-75. Human cav 1 is encoded on chromosome 7q31.1, and there are two isoforms, product of different mRNAs, α and β. Cav 1 α has 31 more amino acids in the N-terminus than cav 1 β 76,77. Analyses of the *CAV1* gene showed 30 CpG sites distributed in two promoter regions containing 7 and 28 CpG sites, respectively. Hypermethylation of the first three sites was strongly associated with decreased expression of cav 1 in SCLC, breast cancer and human T-leukemia cell lines, and neuronal cells 47,58,78,79. Furthermore, the* CAV1* promoter has two ETS binding sites and single binding sites for GATA6, p53, SP1, E cadherin, 3-hydroxy- 3-methylglutaryl coenzyme-A-synthase-1 (HMGCS-1), and CD44. Interestingly, the *CAV1* promoter also has sterol regulatory elements (SREs) 80,81. In fact, cav 1 directly binds cholesterol, a fundamental component of the MR; this may indicate that the role of cav 1 in cholesterol trafficking into the PM and mitochondria is linked with its function in MR signal transduction and metabolic regulation 81-84. Other regulatory mechanisms like loss of heterozygosity have been described within chromosome 7q31.1 85, but there is no evidence for deletions of the *CAV1* gene in human tumors 86.

##### Micro RNA and Long Non-coding RNA regulating cav 1

**Figure 3:**
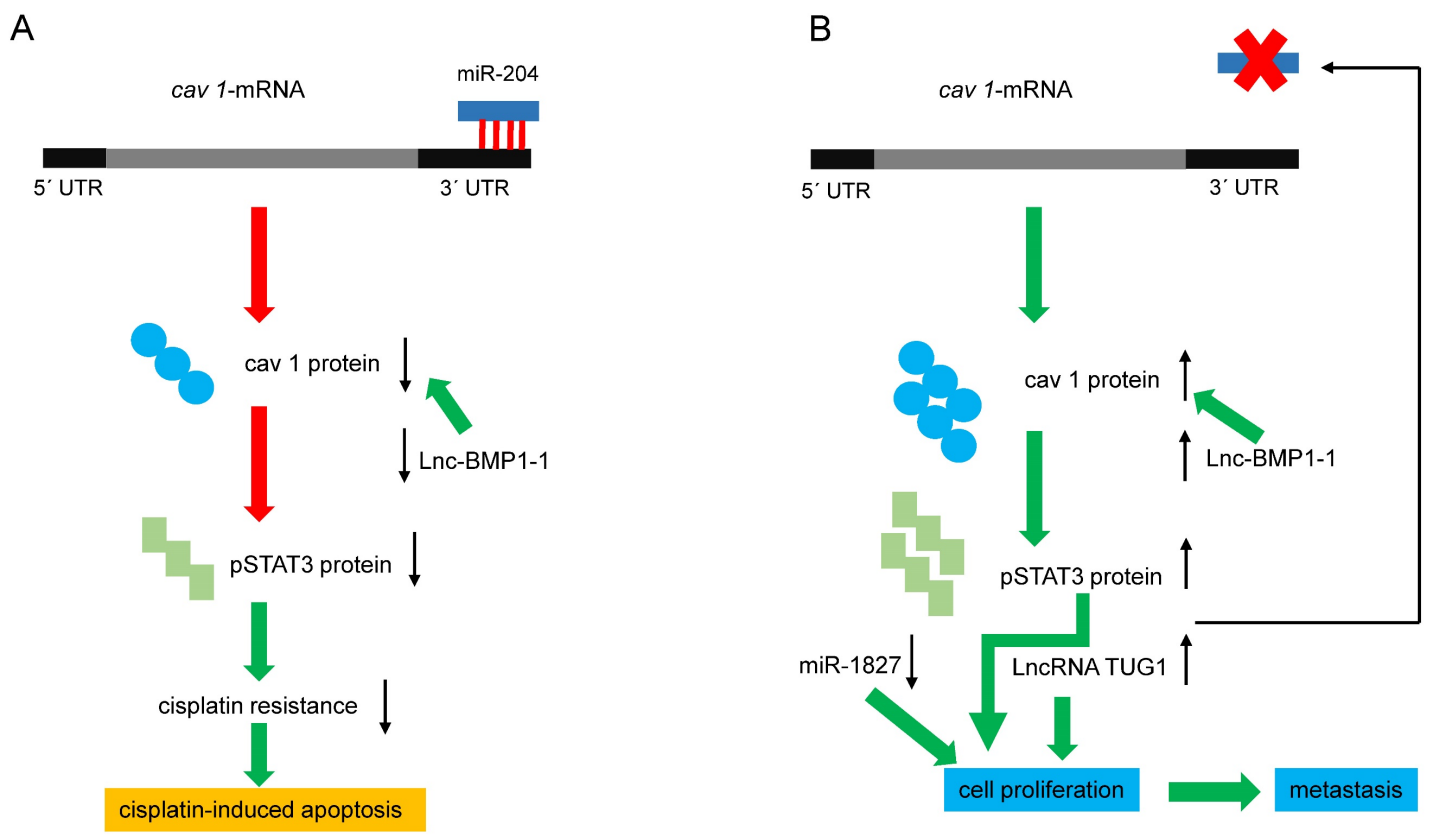
** Regulation of cav 1 expression by miRNAs and LncRNAs. (A)** miRNA-204 downregulates cav 1-mRNA, decreasing cav 1 protein, pSTAT3, and cisplatin resistance. Lnc-BMP1-1 decreases *cav1* transcription, favoring apoptosis. **(B)** Reducing miRNA-204 levels, increases cav 1 and pSTAT3, and consequently, cell proliferation and invasion are favored. On the other hand, pSTAT3 blocks the expression of the miRNA, generating a positive feedback loop. Diminished or increased levels of miR-1827 and LncRNA-TUG1 respectively, favoring cell proliferation and metastasis. Red arrows indicate negative effects, green ones, activating effects.

Recent reports have clarified the mechanisms underlying the positive effect of reducing *CAV 1* expression on cisplatin cell damage in human LC cells. Silencing *CAV1* in A549 cells, enhanced cisplatin‐mediated mitochondrial apoptotic signaling 87. Conversely, a higher expression of microRNA-204 (miR‐204) in A549 and PC9 cells reduces cisplatin resistance, which was related to decreased *CAV1* expression 88. The 3´ UTR of cav 1-mRNA contains a conserved 5´ AAGGGAA 3´ site targeted by miR-204 89, and *CAV1* expression is negatively modulated by miR-204 (figure 3A). The phosphorylation of AKT and Bad can be further suppressed by the miR-204/cav 1 pathway, promoting cisplatin-induced apoptosis by silencing Bcl-2 and Bcl-xl 88. Moreover, the human gene MIR204, encodes a 110 bp pre-miR-204 stem-loop located in intron 6 of transient receptor potential nonselective cation channel, subfamily M, member 3 (TRPM3) in the locus 9q21.12-q21.13 89-91. Both genes are co-transcribed in clear renal carcinoma cells, and miR-204 negatively regulates the translation of TRPM3 by binding to its 3´UTR. Interestingly, the expression of TRPM3 also requires another direct target of miR-204, cav 1: in fact, knocking down cav 1 reduced the expression of TRPM3 91. Since TRPM3 regulates autophagy, the previous results imply that cav 1 modulates this cellular process through another mechanism. The expression of miR-204 is regulated by DNA methylation, long non-coding RNAs (LncRNAs), and transcription factors. LncRNA taurine upregulated 1 (LncRNA TUG1) like DNA methylation blocks the expression of miR-204, whereas the transcription factors Pax6 and STAT3 activate and inhibit its expression, respectively 90. As mentioned previously, silencing *CAV1* gene in human lung carcinoma cells decreases activated pSTAT3, suggesting that high levels of cav 1 could increase pSTAT3 and thereby block the expression of miR-204, as described in renal cancer cells (figure 3B). Using functional and bioinformatics analyses, Guo and coworkers recently identified that microRNA-1827 (miR-1827) directly targets cav 1. In this context, miR-1827 level was decreased in NSCLC tissues and A549 cells, and decreased miR-1827 levels in a manner directly correlated with metastasis. Restoring miR-1827 prevented anoikis resistance, suppressed cell viability, and induced apoptosis in A549 cells, whereas over-expression of cav 1 attenuated these effects 91.

In addition to TUG1, other LncRNAs, such as LET 93 and TARID 94, also play crucial roles in LC tumorigenesis 92. TUG1 is an LncRNA with 6.7-kb nucleotides located at chromosome 22q12 95; this gene is overexpressed in several other types of cancer 96-100. TUG1 shows higher expression in lung tumors than in adjacent tissue, and its expression in LC cell lines favors proliferation and migration and inhibits apoptosis 101 (figure 3B). A recent report found that TUG1 is among the most expressed LncRNAs in LC and may be a biomarker of lung neoplasms. Other LncRNAs, such as PTENP1 and UCA1, could be biomarkers of NSCLC and LUAD, respectively 102. Serum levels of TUG1 provide a high diagnostic value for NSCLC and LUAD 103,104, so it has been proposed as a therapeutic target in SCLC 105.

Lnc-BMP1-1 is another LncRNA related to cav 1 in LC. This gene is transcribed from the intron area of surfactant protein C; thus, highly expressed in the lung. Recent studies show that Lnc-BMP1-1 might promote the transcription of *CAV1* by downregulating the expression of histone deacetylase 2 106 (figure 3A). Lnc-BMP1-1 expression was decreased in LC tissues, especially in patients with a history of cigarette smoking; therefore, it is logical to expect a lower expression of *CAV1* in those patients. Interestingly, it is known that oxidative stress induced by cigarette smoke causes lung tumorigenesis, and smokers are more prone to have lower expression of Lnc-BMP1-1 and cav 1 106. Hence, the following question arises: Does Lnc-BMP1-1 promote the tumor-suppressing effects of cav 1? Considering that cav 1 prevents hydrogen peroxide-induced oxidative damage to lung carcinoma cells 107, and higher cav 1 expression enhances the sensitivity of A549 cells to the anti-cancer drug doxorubicin 106, it is evident that downregulating *CAV1* could be unfavorable for NSCLC. In this context, it was reported that the anti-cancer drug cordycepin is involved in the JNK/Foxo3a axis, which in turn, activates the Bax/caspase-3-mediated pathway. Since cav1 signaling directly targets the Bax/caspase-3-apoptosis mediated pathway, this may be the mechanism whereby cav 1 causes cell death of A549 cells. 108. Therefore, a second question arises: How does NSCLC avoid the negative effects exerted by Lnc-BMP1-1 on *CAV1* expression? The answer still is beyond the currently available experimental data, but clarifying this paradox will be important for LC therapy.

#### Caveolin 2 (cav 2) in LC

The importance of cav 2 in cancer pathogenesis was recognized almost at the same time as that of cav 1. Increased *CAV2* expression is related to poor prognosis of pancreatic cancer both *in vivo* and *in vitro*. The mechanism involves a specific genetic variant that impairs miR-548s binding to the 3´UTR, affecting the expression of genes related to focal adhesion and extracellular matrix organization 109. Regarding LC, only a few reports have implicated cav 2 in cancer progression. Murine lung carcinoma cell lines implanted into cav 2-KO mice were unable to grow, and tumor size was dramatically reduced two weeks after implantation 110,111. In these murine models, cav 2 deficiency triggered an anti-tumor immune response and impaired tumor angiogenesis. Host cav 2 deficiency reduced tumor cell proliferation in the late stages of the implanted tumor's development, when defective neovascularization is essential for supporting tumor growth. In fact, cav 2 knockout mice showed more necrotic than apoptotic cell death within the implanted tumors 111. Interestingly, cav 2 deficiency significantly increased anti-angiogenic thrombospondin 1 (TSP1) mRNA levels, with the concomitant decrease in endothelial nitric oxide synthase (eNOS) phosphorylation and a decrease in pro-angiogenic factors such as vascular endothelial growth factor-A (AVEGF-A).

### Flotillins in LC

Flotillin 1 and flotillin 2 (flot 1 and flot 2) are MR-associated proteins involved in several cellular functions, such as signaling, endocytosis, and other cytoskeleton-related roles. The expression of both proteins is interdependent, i.e., the downregulation of flot 2 severely reduces flot 1 expression 112. Both flot 1 and 2 are associated with LC 112-115. Data from knockdown models and flot overexpression in cell culture indicate that overexpression of flot 1 inhibits apoptosis and enhances the malignant behavior of LUAD by promoting cell growth, invasion, and migration. The expression of several cell cycle regulatory markers, such as cyclin D kinase 2 (CDK2), cyclin E, and cyclin D1, were significantly increased, whereas p16 expression was reduced 114. Epithelial-mesenchymal transition is also favored by overexpression flot 1, and it is mediated by higher Akt phosphorylation of and FOXO3a downregulation 114. Interestingly, it has been reported that, in NSCLC, overexpression of cytoskeleton protein 4.1N significantly reduces the expression of flot 1, avoiding its carcinogenic effects through lower activation of the Wnt/β-catenin/c-Myc pathway and higher E-cadherin expression 115. Nevertheless, increased flot 2 expression was significantly associated with higher EGFR expression in NSCLC compared to the non-cancerous lung control tissues 116. EGFR overexpression and EGFR mutations play a key role in NSCLC, mainly by resistance to its degradation. Clathrin-independent EGFR endocytosis is mediated by flot 1 and MR, and induces the lysosomal degradation of EGFR 117. Taken together, these data indicate that flot 1 is essential for tumor suppressor in NSCLC. Therefore, flotillins seem to have oncogenic and tumor suppressor effects similar to those of cav 1.

## Final remarks

Cancer cells can evade apoptosis through well-known mechanisms involving defects in cell signaling or apoptosis mediators. Since MRs participate in cell signaling and the activation of apoptosis, the proteins that determine the assemblage and proper functioning of MR, such as caveolins and flotillins, are targets for regulating these physiological processes. However, the fact that these proteins act both as tumor suppressors and oncoproteins is intriguing and still unclarified. Beyond caveolins and flotillins, this phenomenon is extensive to other MR “scaffolding” proteins, for example, several MR proteins with the MARVEL (MAL and related proteins for vesicle trafficking and membrane link) domain. However, MARVEL proteins differ from caveolins and flotillins because they cross the membrane four times, with extracellular, transmembrane, and intracellular segments. Myelin and lymphocyte protein (MAL), MAL2, MAL like protein (MALL, formerly BENE) are involved in several human cancers 23,118,119, either promoting or suppressing tumor growth and metastasis 120-122. The effect of these proteins on cancer, whether beneficial or detrimental, depends on epigenetic mechanisms. Therefore, considering the initial proposal by doctors Weinberg and Hanahan 123, we must continue searching for common ground between the processes and mediators driving cell transformation.
